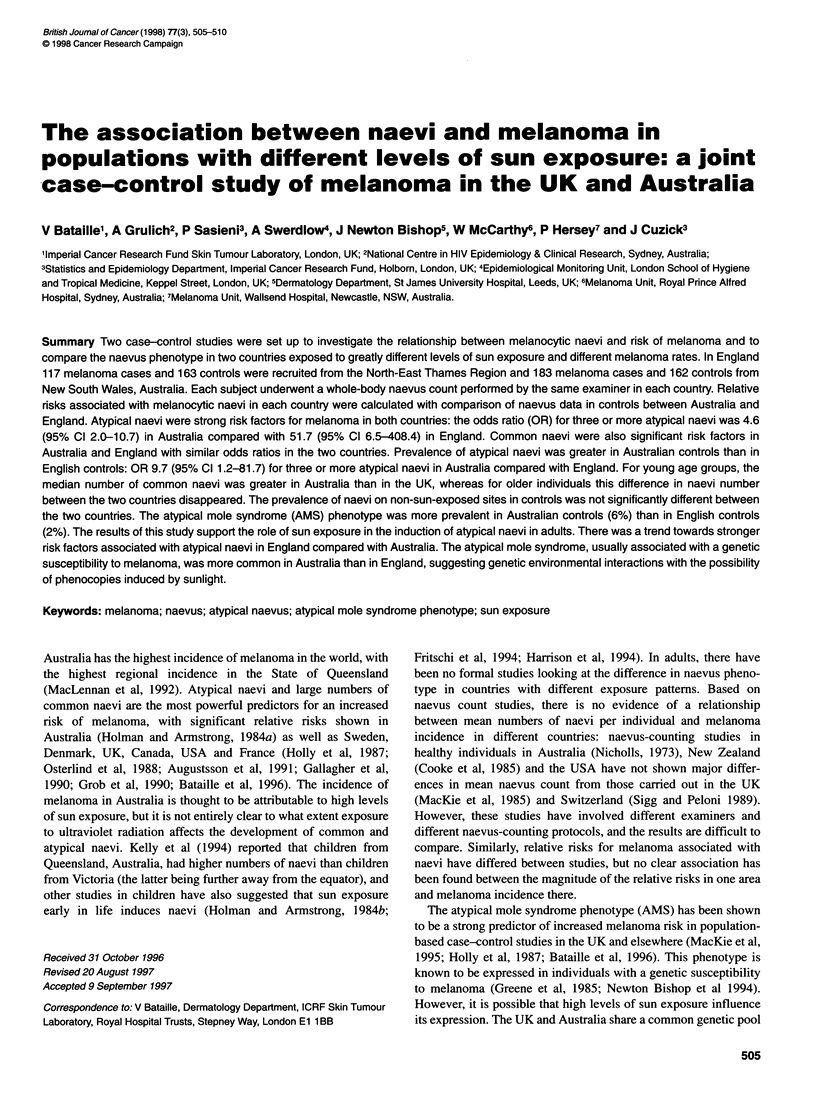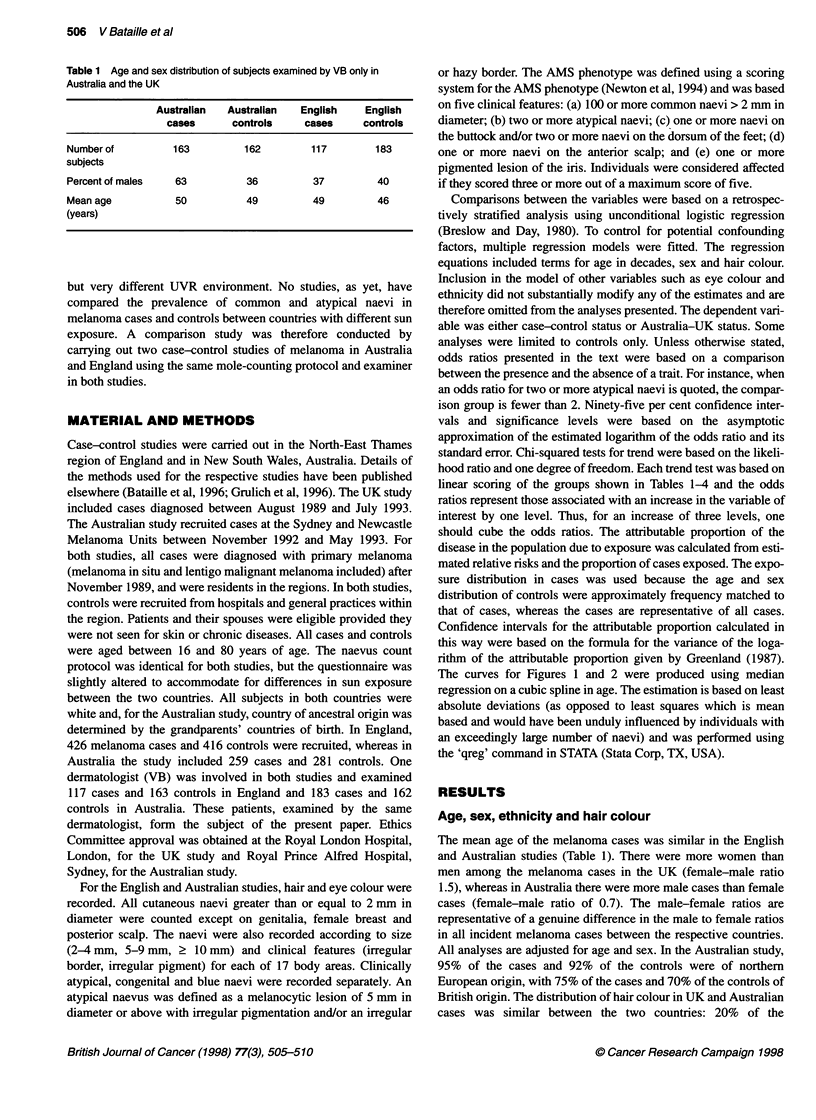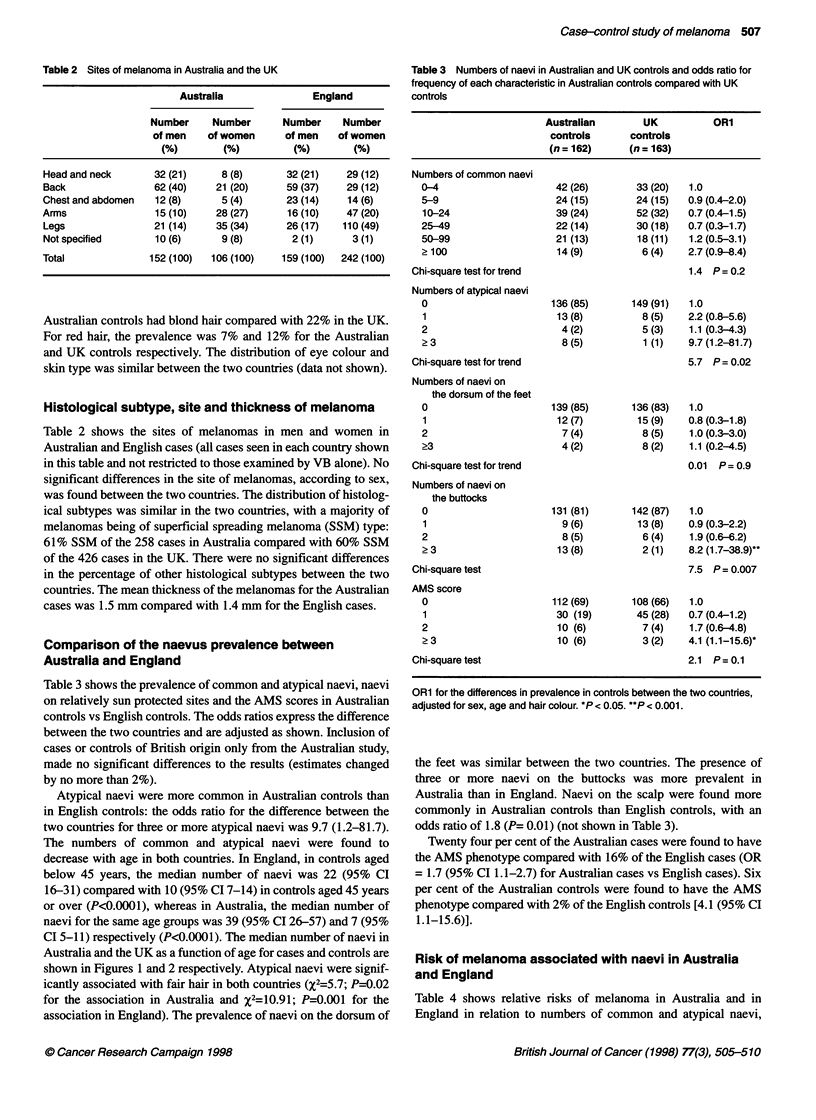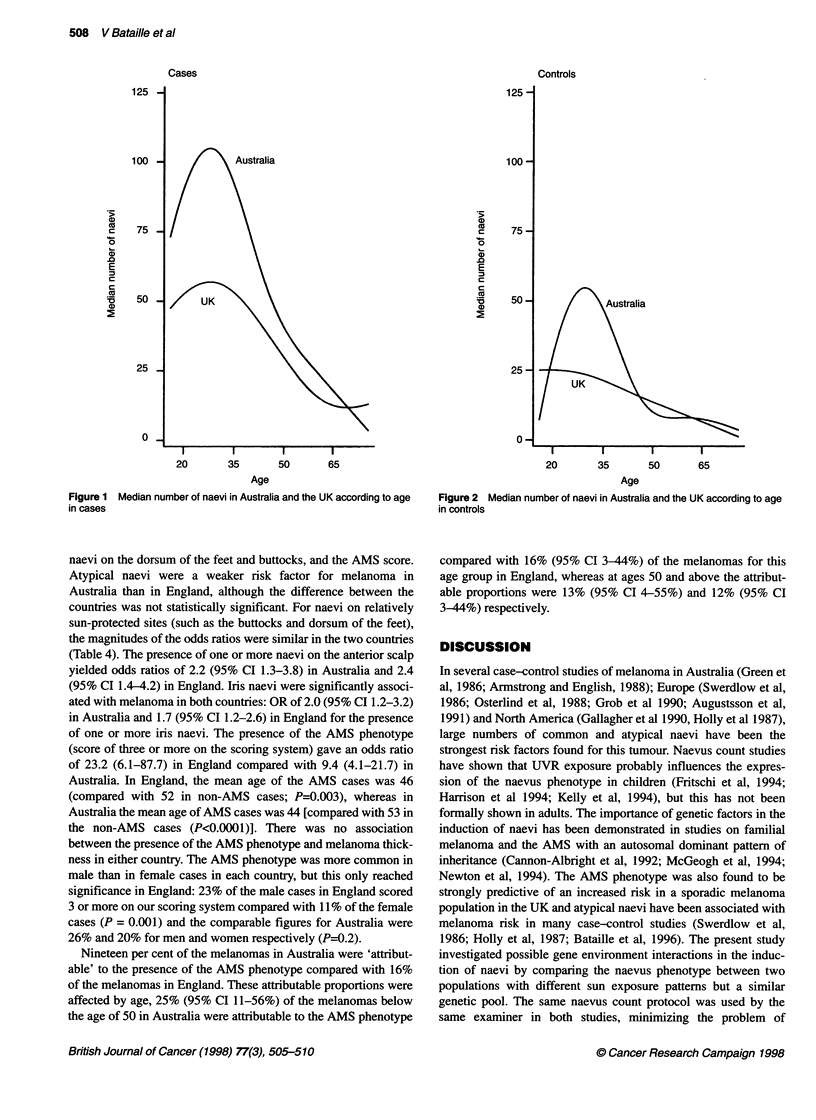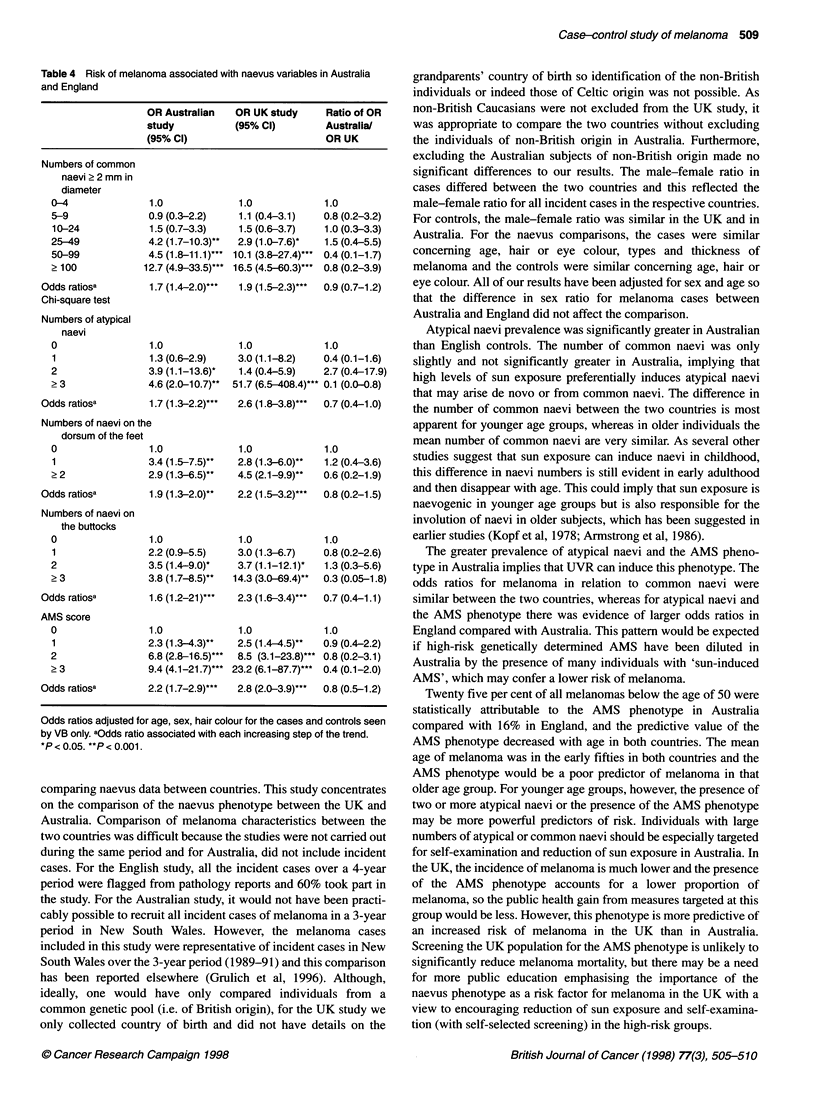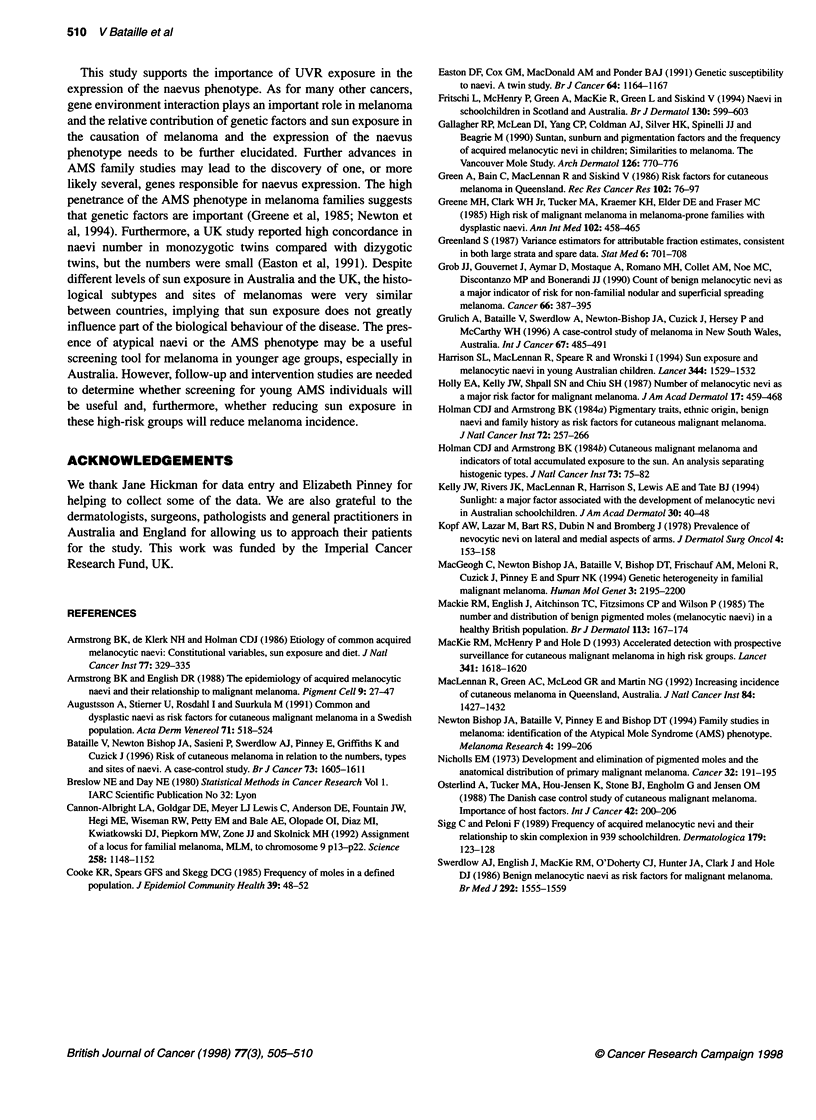# The association between naevi and melanoma in populations with different levels of sun exposure: a joint case-control study of melanoma in the UK and Australia.

**DOI:** 10.1038/bjc.1998.81

**Published:** 1998

**Authors:** V. Bataille, A. Grulich, P. Sasieni, A. Swerdlow, J. Newton Bishop, W. McCarthy, P. Hersey, J. Cuzick

**Affiliations:** Imperial Cancer Research Fund Skin Tumour Laboratory, London, UK.

## Abstract

Two case-control studies were set up to investigate the relationship between melanocytic naevi and risk of melanoma and to compare the naevus phenotype in two countries exposed to greatly different levels of sun exposure and different melanoma rates. In England 117 melanoma cases and 163 controls were recruited from the North-East Thames Region and 183 melanoma cases and 162 controls from New South Wales, Australia. Each subject underwent a whole-body naevus count performed by the same examiner in each country. Relative risks associated with melanocytic naevi in each country were calculated with comparison of naevus data in controls between Australia and England. Atypical naevi were strong risk factors for melanoma in both countries: the odds ratio (OR) for three or more atypical naevi was 4.6 (95% CI 2.0-10.7) in Australia compared with 51.7 (95% CI 6.5-408.4) in England. Common naevi were also significant risk factors in Australia and England with similar odds ratios in the two countries. Prevalence of atypical naevi was greater in Australian controls than in English controls: OR 9.7 (95% CI 1.2-81.7) for three or more atypical naevi in Australia compared with England. For young age groups, the median number of common naevi was greater in Australia than in the UK, whereas for older individuals this difference in naevi number between the two countries disappeared. The prevalence of naevi on non-sun-exposed sites in controls was not significantly different between the two countries. The atypical mole syndrome (AMS) phenotype was more prevalent in Australian controls (6%) than in English controls (2%). The results of this study support the role of sun exposure in the induction of atypical naevi in adults. There was a trend towards stronger risk factors associated with atypical naevi in England compared with Australia. The atypical mole syndrome, usually associated with a genetic susceptibility to melanoma, was more common in Australia than in England, suggesting genetic environmental interactions with the possibility of phenocopies induced by sunlight.


British Journal of Cancer (1998) 77(3), 505–510
© 1998 Cancer Research Campaign

### The association between naevi and melanoma in populations with different levels of sun exposure: a joint case–control study of melanoma in the UK and Australia

V Bataille[1], A Grulich[2], P Sasieni[3], A Swerdlow[4], J Newton Bishop[5], W McCarthy[6], P Hersey[7] and J Cuzick[3]

[1]Imperial Cancer Research Fund Skin Tumour Laboratory, London, UK; [2]National Centre in HIV Epidemiology & Clinical Research, Sydney, Australia; [3]Statistics and Epidemiology Department, Imperial Cancer Research Fund, Holborn, London, UK; [4]Epidemiological Monitoring Unit, London School of Hygiene and Tropical Medicine, Keppel Street, London, UK; [5]Dermatology Department, St James University Hospital, Leeds, UK; [6]Melanoma Unit, Royal Prince Alfred Hospital, Sydney, Australia; [7]Melanoma Unit, Wallsend Hospital, Newcastle, NSW, Australia.

**Summary** Two case–control studies were set up to investigate the relationship between melanocytic naevi and risk of melanoma and to compare the naevus phenotype in two countries exposed to greatly different levels of sun exposure and different melanoma rates. In England 117 melanoma cases and 163 controls were recruited from the North-East Thames Region and 183 melanoma cases and 162 controls from New South Wales, Australia. Each subject underwent a whole-body naevus count performed by the same examiner in each country. Relative risks associated with melanocytic naevi in each country were calculated with comparison of naevus data in controls between Australia and England. Atypical naevi were strong risk factors for melanoma in both countries: the odds ratio (OR) for three or more atypical naevi was 4.6 (95% CI 2.0–10.7) in Australia compared with 51.7 (95% CI 6.5–408.4) in England. Common naevi were also significant risk factors in Australia and England with similar odds ratios in the two countries. Prevalence of atypical naevi was greater in Australian controls than in English controls: OR 9.7 (95% CI 1.2–81.7) for three or more atypical naevi in Australia compared with England. For young age groups, the median number of common naevi was greater in Australia than in the UK, whereas for older individuals this difference in naevi number between the two countries disappeared. The prevalence of naevi on non-sun-exposed sites in controls was not significantly different between the two countries. The atypical mole syndrome (AMS) phenotype was more prevalent in Australian controls (6%) than in English controls (2%). The results of this study support the role of sun exposure in the induction of atypical naevi in adults. There was a trend towards stronger risk factors associated with atypical naevi in England compared with Australia. The atypical mole syndrome, usually associated with a genetic susceptibility to melanoma, was more common in Australia than in England, suggesting genetic environmental interactions with the possibility of phenocopies induced by sunlight.

**Keywords:** melanoma; naevus; atypical naevus; atypical mole syndrome phenotype; sun exposure

Australia has the highest incidence of melanoma in the world, with the highest regional incidence in the State of Queensland (MacLennan et al, 1992). Atypical naevi and large numbers of common naevi are the most powerful predictors for an increased risk of melanoma, with significant relative risks shown in Australia (Holman and Armstrong, 1984*a*) as well as Sweden, Denmark, UK, Canada, USA and France (Holly et al, 1987; Osterlind et al, 1988; Augustsson et al, 1991; Gallagher et al, 1990; Grob et al, 1990; Bataille et al, 1996). The incidence of melanoma in Australia is thought to be attributable to high levels of sun exposure, but it is not entirely clear to what extent exposure to ultraviolet radiation affects the development of common and atypical naevi. Kelly et al (1994) reported that children from Queensland, Australia, had higher numbers of naevi than children from Victoria (the latter being further away from the equator), and other studies in children have also suggested that sun exposure early in life induces naevi (Holman and Armstrong, 1984*b*; Fritschi et al, 1994; Harrison et al, 1994). In adults, there have been no formal studies looking at the difference in naevus phenotype in countries with different exposure patterns. Based on naevus count studies, there is no evidence of a relationship between mean numbers of naevi per individual and melanoma incidence in different countries: naevus-counting studies in healthy individuals in Australia (Nicholls, 1973), New Zealand (Cooke et al, 1985) and the USA have not shown major differences in mean naevus count from those carried out in the UK (MacKie et al, 1985) and Switzerland (Sigg and Peloni 1989). However, these studies have involved different examiners and different naevus-counting protocols, and the results are difficult to compare. Similarly, relative risks for melanoma associated with naevi have differed between studies, but no clear association has been found between the magnitude of the relative risks in one area and melanoma incidence there.

*Received 31 October 1996*
*Revised 20 August 1997*
*Accepted 9 September 1997*

*Correspondence to:* V Bataille, Dermatology Department, ICRF Skin Tumour Laboratory, Royal Hospital Trusts, Stepney Way, London E1 1BB

The atypical mole syndrome phenotype (AMS) has been shown to be a strong predictor of increased melanoma risk in population-based case–control studies in the UK and elsewhere (MacKie et al, 1995; Holly et al, 1987; Bataille et al, 1996). This phenotype is known to be expressed in individuals with a genetic susceptibility to melanoma (Greene et al, 1985; Newton Bishop et al 1994). However, it is possible that high levels of sun exposure influence its expression. The UK and Australia share a common genetic pool

Table 1 Age and sex distribution of subjects examined by VB only in Australia and the UK

| | Australian cases | Australian controls | English cases | English controls |
|---|---|---|---|---|
| Number of subjects | 163 | 162 | 117 | 183 |
| Percent of males | 63 | 36 | 37 | 40 |
| Mean age (years) | 50 | 49 | 49 | 46 |

but very different UVR environment. No studies, as yet, have compared the prevalence of common and atypical naevi in melanoma cases and controls between countries with different sun exposure. A comparison study was therefore conducted by carrying out two case–control studies of melanoma in Australia and England using the same mole-counting protocol and examiner in both studies.

### MATERIAL AND METHODS

Case–control studies were carried out in the North-East Thames region of England and in New South Wales, Australia. Details of the methods used for the respective studies have been published elsewhere (Bataille et al, 1996; Grulich et al, 1996). The UK study included cases diagnosed between August 1989 and July 1993. The Australian study recruited cases at the Sydney and Newcastle Melanoma Units between November 1992 and May 1993. For both studies, all cases were diagnosed with primary melanoma (melanoma in situ and lentigo malignant melanoma included) after November 1989, and were residents in the regions. In both studies, controls were recruited from hospitals and general practices within the region. Patients and their spouses were eligible provided they were not seen for skin or chronic diseases. All cases and controls were aged between 16 and 80 years of age. The naevus count protocol was identical for both studies, but the questionnaire was slightly altered to accommodate for differences in sun exposure between the two countries. All subjects in both countries were white and, for the Australian study, country of ancestral origin was determined by the grandparents' countries of birth. In England, 426 melanoma cases and 416 controls were recruited, whereas in Australia the study included 259 cases and 281 controls. One dermatologist (VB) was involved in both studies and examined 117 cases and 163 controls in England and 183 cases and 162 controls in Australia. These patients, examined by the same dermatologist, form the subject of the present paper. Ethics Committee approval was obtained at the Royal London Hospital, London, for the UK study and Royal Prince Alfred Hospital, Sydney, for the Australian study.

For the English and Australian studies, hair and eye colour were recorded. All cutaneous naevi greater than or equal to 2 mm in diameter were counted except on genitalia, female breast and posterior scalp. The naevi were also recorded according to size (2–4 mm, 5–9 mm, ≥ 10 mm) and clinical features (irregular border, irregular pigment) for each of 17 body areas. Clinically atypical, congenital and blue naevi were recorded separately. An atypical naevus was defined as a melanocytic lesion of 5 mm in diameter or above with irregular pigmentation and/or an irregular or hazy border. The AMS phenotype was defined using a scoring system for the AMS phenotype (Newton et al, 1994) and was based on five clinical features: (a) 100 or more common naevi > 2 mm in diameter; (b) two or more atypical naevi; (c) one or more naevi on the buttock and/or two or more naevi on the dorsum of the feet; (d) one or more naevi on the anterior scalp; and (e) one or more pigmented lesion of the iris. Individuals were considered affected if they scored three or more out of a maximum score of five.

Comparisons between the variables were based on a retrospectively stratified analysis using unconditional logistic regression (Breslow and Day, 1980). To control for potential confounding factors, multiple regression models were fitted. The regression equations included terms for age in decades, sex and hair colour. Inclusion in the model of other variables such as eye colour and ethnicity did not substantially modify any of the estimates and are therefore omitted from the analyses presented. The dependent variable was either case–control status or Australia–UK status. Some analyses were limited to controls only. Unless otherwise stated, odds ratios presented in the text were based on a comparison between the presence and the absence of a trait. For instance, when an odds ratio for two or more atypical naevi is quoted, the comparison group is fewer than 2. Ninety-five per cent confidence intervals and significance levels were based on the asymptotic approximation of the estimated logarithm of the odds ratio and its standard error. Chi-squared tests for trend were based on the likelihood ratio and one degree of freedom. Each trend test was based on linear scoring of the groups shown in Tables 1–4 and the odds ratios represent those associated with an increase in the variable of interest by one level. Thus, for an increase of three levels, one should cube the odds ratios. The attributable proportion of the disease in the population due to exposure was calculated from estimated relative risks and the proportion of cases exposed. The exposure distribution in cases was used because the age and sex distribution of controls were approximately frequency matched to that of cases, whereas the cases are representative of all cases. Confidence intervals for the attributable proportion calculated in this way were based on the formula for the variance of the logarithm of the attributable proportion given by Greenland (1987). The curves for Figures 1 and 2 were produced using median regression on a cubic spline in age. The estimation is based on least absolute deviations (as opposed to least squares which is mean based and would have been unduly influenced by individuals with an exceedingly large number of naevi) and was performed using the 'qreg' command in STATA (Stata Corp, TX, USA).

### RESULTS

### Age, sex, ethnicity and hair colour

The mean age of the melanoma cases was similar in the English and Australian studies (Table 1). There were more women than men among the melanoma cases in the UK (female–male ratio 1.5), whereas in Australia there were more male cases than female cases (female–male ratio of 0.7). The male–female ratios are representative of a genuine difference in the male to female ratios in all incident melanoma cases between the respective countries. All analyses are adjusted for age and sex. In the Australian study, 95% of the cases and 92% of the controls were of northern European origin, with 75% of the cases and 70% of the controls of British origin. The distribution of hair colour in UK and Australian cases was similar between the two countries: 20% of the

© Cancer Research Campaign 1998

**Table 2** Sites of melanoma in Australia and the UK

| | Australia | | England | |
|---|---|---|---|---|
| | Number of men (%) | Number of women (%) | Number of men (%) | Number of women (%) |
| Head and neck | 32 (21) | 8 (8) | 32 (21) | 29 (12) |
| Back | 62 (40) | 21 (20) | 59 (37) | 29 (12) |
| Chest and abdomen | 12 (8) | 5 (4) | 23 (14) | 14 (6) |
| Arms | 15 (10) | 28 (27) | 16 (10) | 47 (20) |
| Legs | 21 (14) | 35 (34) | 26 (17) | 110 (49) |
| Not specified | 10 (6) | 9 (8) | 2 (1) | 3 (1) |
| Total | 152 (100) | 106 (100) | 159 (100) | 242 (100) |

Australian controls had blond hair compared with 22% in the UK. For red hair, the prevalence was 7% and 12% for the Australian and UK controls respectively. The distribution of eye colour and skin type was similar between the two countries (data not shown).

### Histological subtype, site and thickness of melanoma

Table 2 shows the sites of melanomas in men and women in Australian and English cases (all cases seen in each country shown in this table and not restricted to those examined by VB alone). No significant differences in the site of melanomas, according to sex, was found between the two countries. The distribution of histological subtypes was similar in the two countries, with a majority of melanomas being of superficial spreading melanoma (SSM) type: 61% SSM of the 258 cases in Australia compared with 60% SSM of the 426 cases in the UK. There were no significant differences in the percentage of other histological subtypes between the two countries. The mean thickness of the melanomas for the Australian cases was 1.5 mm compared with 1.4 mm for the English cases.

### Comparison of the naevus prevalence between Australia and England

Table 3 shows the prevalence of common and atypical naevi, naevi on relatively sun protected sites and the AMS scores in Australian controls vs English controls. The odds ratios express the difference between the two countries and are adjusted as shown. Inclusion of cases or controls of British origin only from the Australian study, made no significant differences to the results (estimates changed by no more than 2%).

Atypical naevi were more common in Australian controls than in English controls: the odds ratio for the difference between the two countries for three or more atypical naevi was 9.7 (1.2–81.7). The numbers of common and atypical naevi were found to decrease with age in both countries. In England, in controls aged below 45 years, the median number of naevi was 22 (95% CI 16–31) compared with 10 (95% CI 7–14) in controls aged 45 years or over ($P$<0.0001), whereas in Australia, the median number of naevi for the same age groups was 39 (95% CI 26–57) and 7 (95% CI 5–11) respectively ($P$<0.0001). The median number of naevi in Australia and the UK as a function of age for cases and controls are shown in Figures 1 and 2 respectively. Atypical naevi were significantly associated with fair hair in both countries ($\chi^2$=5.7; $P$=0.02 for the association in Australia and $\chi^2$=10.91; $P$=0.001 for the association in England). The prevalence of naevi on the dorsum of the feet was similar between the two countries. The presence of three or more naevi on the buttocks was more prevalent in Australia than in England. Naevi on the scalp were found more commonly in Australian controls than English controls, with an odds ratio of 1.8 ($P$= 0.01) (not shown in Table 3).

**Table 3** Numbers of naevi in Australian and UK controls and odds ratio for frequency of each characteristic in Australian controls compared with UK controls

| | Australian controls ($n$ = 162) | UK controls ($n$ = 163) | OR1 |
|---|---|---|---|
| Numbers of common naevi | | | |
| 0–4 | 42 (26) | 33 (20) | 1.0 |
| 5–9 | 24 (15) | 24 (15) | 0.9 (0.4–2.0) |
| 10–24 | 39 (24) | 52 (32) | 0.7 (0.4–1.5) |
| 25–49 | 22 (14) | 30 (18) | 0.7 (0.3–1.7) |
| 50–99 | 21 (13) | 18 (11) | 1.2 (0.5–3.1) |
| ≥ 100 | 14 (9) | 6 (4) | 2.7 (0.9–8.4) |
| Chi-square test for trend | | | 1.4 $P$ = 0.2 |
| Numbers of atypical naevi | | | |
| 0 | 136 (85) | 149 (91) | 1.0 |
| 1 | 13 (8) | 8 (5) | 2.2 (0.8–5.6) |
| 2 | 4 (2) | 5 (3) | 1.1 (0.3–4.3) |
| ≥ 3 | 8 (5) | 1 (1) | 9.7 (1.2–81.7) |
| Chi-square test for trend | | | 5.7 $P$ = 0.02 |
| Numbers of naevi on the dorsum of the feet | | | |
| 0 | 139 (85) | 136 (83) | 1.0 |
| 1 | 12 (7) | 15 (9) | 0.8 (0.3–1.8) |
| 2 | 7 (4) | 8 (5) | 1.0 (0.3–3.0) |
| ≥3 | 4 (2) | 8 (2) | 1.1 (0.2–4.5) |
| Chi-square test for trend | | | 0.01 $P$ = 0.9 |
| Numbers of naevi on the buttocks | | | |
| 0 | 131 (81) | 142 (87) | 1.0 |
| 1 | 9 (6) | 13 (8) | 0.9 (0.3–2.2) |
| 2 | 8 (5) | 6 (4) | 1.9 (0.6–6.2) |
| ≥ 3 | 13 (8) | 2 (1) | 8.2 (1.7–38.9)** |
| Chi-square test | | | 7.5 $P$ = 0.007 |
| AMS score | | | |
| 0 | 112 (69) | 108 (66) | 1.0 |
| 1 | 30 (19) | 45 (28) | 0.7 (0.4–1.2) |
| 2 | 10 (6) | 7 (4) | 1.7 (0.6–4.8) |
| ≥ 3 | 10 (6) | 3 (2) | 4.1 (1.1–15.6)* |
| Chi-square test | | | 2.1 $P$ = 0.1 |

OR1 for the differences in prevalence in controls between the two countries, adjusted for sex, age and hair colour. *$P$ < 0.05. **$P$ < 0.001.

Twenty four per cent of the Australian cases were found to have the AMS phenotype compared with 16% of the English cases (OR = 1.7 (95% CI 1.1–2.7) for Australian cases vs English cases). Six per cent of the Australian controls were found to have the AMS phenotype compared with 2% of the English controls [4.1 (95% CI 1.1–15.6)].

### Risk of melanoma associated with naevi in Australia and England

Table 4 shows relative risks of melanoma in Australia and in England in relation to numbers of common and atypical naevi,

© Cancer Research Campaign 1998

**Figure 1** Median number of naevi in Australia and the UK according to age in cases

**Figure 2** Median number of naevi in Australia and the UK according to age in controls naevi on the dorsum of the feet and buttocks, and the AMS score. Atypical naevi were a weaker risk factor for melanoma in Australia than in England, although the difference between the countries was not statistically significant. For naevi on relatively sun-protected sites (such as the buttocks and dorsum of the feet), the magnitudes of the odds ratios were similar in the two countries (Table 4). The presence of one or more naevi on the anterior scalp yielded odds ratios of 2.2 (95% CI 1.3–3.8) in Australia and 2.4 (95% CI 1.4–4.2) in England. Iris naevi were significantly associated with melanoma in both countries: OR of 2.0 (95% CI 1.2–3.2) in Australia and 1.7 (95% CI 1.2–2.6) in England for the presence of one or more iris naevi. The presence of the AMS phenotype (score of three or more on the scoring system) gave an odds ratio of 23.2 (6.1–87.7) in England compared with 9.4 (4.1–21.7) in Australia. In England, the mean age of the AMS cases was 46 (compared with 52 in non-AMS cases; $P$=0.003), whereas in Australia the mean age of AMS cases was 44 [compared with 53 in the non-AMS cases ($P$<0.0001)]. There was no association between the presence of the AMS phenotype and melanoma thickness in either country. The AMS phenotype was more common in male than in female cases in each country, but this only reached significance in England: 23% of the male cases in England scored 3 or more on our scoring system compared with 11% of the female cases ($P$ = 0.001) and the comparable figures for Australia were 26% and 20% for men and women respectively ($P$=0.2).

Nineteen per cent of the melanomas in Australia were 'attributable' to the presence of the AMS phenotype compared with 16% of the melanomas in England. These attributable proportions were affected by age, 25% (95% CI 11–56%) of the melanomas below the age of 50 in Australia were attributable to the AMS phenotype compared with 16% (95% CI 3–44%) of the melanomas for this age group in England, whereas at ages 50 and above the attributable proportions were 13% (95% CI 4–55%) and 12% (95% CI 3–44%) respectively.

### DISCUSSION

In several case–control studies of melanoma in Australia (Green et al, 1986; Armstrong and English, 1988); Europe (Swerdlow et al, 1986; Osterlind et al, 1988; Grob et al 1990; Augustsson et al, 1991) and North America (Gallagher et al 1990, Holly et al 1987), large numbers of common and atypical naevi have been the strongest risk factors found for this tumour. Naevus count studies have shown that UVR exposure probably influences the expression of the naevus phenotype in children (Fritschi et al, 1994; Harrison et al 1994; Kelly et al, 1994), but this has not been formally shown in adults. The importance of genetic factors in the induction of naevi has been demonstrated in studies on familial melanoma and the AMS with an autosomal dominant pattern of inheritance (Cannon-Albright et al, 1992; McGeogh et al, 1994; Newton et al, 1994). The AMS phenotype was also found to be strongly predictive of an increased risk in a sporadic melanoma population in the UK and atypical naevi have been associated with melanoma risk in many case–control studies (Swerdlow et al, 1986; Holly et al, 1987; Bataille et al, 1996). The present study investigated possible gene environment interactions in the induction of naevi by comparing the naevus phenotype between two populations with different sun exposure patterns but a similar genetic pool. The same naevus count protocol was used by the same examiner in both studies, minimizing the problem of

© Cancer Research Campaign 1998

**Table 4** Risk of melanoma associated with naevus variables in Australia and England

| | OR Australian study (95% CI) | OR UK study (95% CI) | Ratio of OR Australia/ OR UK |
|---|---|---|---|
| Numbers of common naevi ≥ 2 mm in diameter | | | |
| 0–4 | 1.0 | 1.0 | 1.0 |
| 5–9 | 0.9 (0.3–2.2) | 1.1 (0.4–3.1) | 0.8 (0.2–3.2) |
| 10–24 | 1.5 (0.7–3.3) | 1.5 (0.6–3.7) | 1.0 (0.3–3.3) |
| 25–49 | 4.2 (1.7–10.3)** | 2.9 (1.0–7.6)* | 1.5 (0.4–5.5) |
| 50–99 | 4.5 (1.8–11.1)*** | 10.1 (3.8–27.4)*** | 0.4 (0.1–1.7) |
| ≥ 100 | 12.7 (4.9–33.5)*** | 16.5 (4.5–60.3)*** | 0.8 (0.2–3.9) |
| Odds ratios[a] Chi-square test | 1.7 (1.4–2.0)*** | 1.9 (1.5–2.3)*** | 0.9 (0.7–1.2) |
| Numbers of atypical naevi | | | |
| 0 | 1.0 | 1.0 | 1.0 |
| 1 | 1.3 (0.6–2.9) | 3.0 (1.1–8.2) | 0.4 (0.1–1.6) |
| 2 | 3.9 (1.1–13.6)* | 1.4 (0.4–5.9) | 2.7 (0.4–17.9) |
| ≥ 3 | 4.6 (2.0–10.7)** | 51.7 (6.5–408.4)*** | 0.1 (0.0–0.8) |
| Odds ratios[a] | 1.7 (1.3–2.2)*** | 2.6 (1.8–3.8)*** | 0.7 (0.4–1.0) |
| Numbers of naevi on the dorsum of the feet | | | |
| 0 | 1.0 | 1.0 | 1.0 |
| 1 | 3.4 (1.5–7.5)** | 2.8 (1.3–6.0)** | 1.2 (0.4–3.6) |
| ≥ 2 | 2.9 (1.3–6.5)** | 4.5 (2.1–9.9)** | 0.6 (0.2–1.9) |
| Odds ratios[a] | 1.9 (1.3–2.0)** | 2.2 (1.5–3.2)*** | 0.8 (0.2–1.5) |
| Numbers of naevi on the buttocks | | | |
| 0 | 1.0 | 1.0 | 1.0 |
| 1 | 2.2 (0.9–5.5) | 3.0 (1.3–6.7) | 0.8 (0.2–2.6) |
| 2 | 3.5 (1.4–9.0)* | 3.7 (1.1–12.1)* | 1.3 (0.3–5.6) |
| ≥ 3 | 3.8 (1.7–8.5)** | 14.3 (3.0–69.4)** | 0.3 (0.05–1.8) |
| Odds ratios[a] | 1.6 (1.2–21)*** | 2.3 (1.6–3.4)*** | 0.7 (0.4–1.1) |
| AMS score | | | |
| 0 | 1.0 | 1.0 | 1.0 |
| 1 | 2.3 (1.3–4.3)** | 2.5 (1.4–4.5)** | 0.9 (0.4–2.2) |
| 2 | 6.8 (2.8–16.5)*** | 8.5 (3.1–23.8)*** | 0.8 (0.2–3.1) |
| ≥ 3 | 9.4 (4.1–21.7)*** | 23.2 (6.1–87.7)*** | 0.4 (0.1–2.0) |
| Odds ratios[a] | 2.2 (1.7–2.9)*** | 2.8 (2.0–3.9)*** | 0.8 (0.5–1.2) |

Odds ratios adjusted for age, sex, hair colour for the cases and controls seen by VB only. [a]Odds ratio associated with each increasing step of the trend. *$P < 0.05$. **$P < 0.001$.

comparing naevus data between countries. This study concentrates on the comparison of the naevus phenotype between the UK and Australia. Comparison of melanoma characteristics between the two countries was difficult because the studies were not carried out during the same period and for Australia, did not include incident cases. For the English study, all the incident cases over a 4-year period were flagged from pathology reports and 60% took part in the study. For the Australian study, it would not have been practicably possible to recruit all incident cases of melanoma in a 3-year period in New South Wales. However, the melanoma cases included in this study were representative of incident cases in New South Wales over the 3-year period (1989–91) and this comparison has been reported elsewhere (Grulich et al, 1996). Although, ideally, one would have only compared individuals from a common genetic pool (i.e. of British origin), for the UK study we only collected country of birth and did not have details on the grandparents' country of birth so identification of the non-British individuals or indeed those of Celtic origin was not possible. As non-British Caucasians were not excluded from the UK study, it was appropriate to compare the two countries without excluding the individuals of non-British origin in Australia. Furthermore, excluding the Australian subjects of non-British origin made no significant differences to our results. The male–female ratio in cases differed between the two countries and this reflected the male–female ratio for all incident cases in the respective countries. For controls, the male–female ratio was similar in the UK and in Australia. For the naevus comparisons, the cases were similar concerning age, hair or eye colour, types and thickness of melanoma and the controls were similar concerning age, hair or eye colour. All of our results have been adjusted for sex and age so that the difference in sex ratio for melanoma cases between Australia and England did not affect the comparison.

Atypical naevi prevalence was significantly greater in Australian than English controls. The number of common naevi was only slightly and not significantly greater in Australia, implying that high levels of sun exposure preferentially induces atypical naevi that may arise de novo or from common naevi. The difference in the number of common naevi between the two countries is most apparent for younger age groups, whereas in older individuals the mean number of common naevi are very similar. As several other studies suggest that sun exposure can induce naevi in childhood, this difference in naevi numbers is still evident in early adulthood and then disappear with age. This could imply that sun exposure is naevogenic in younger age groups but is also responsible for the involution of naevi in older subjects, which has been suggested in earlier studies (Kopf et al, 1978; Armstrong et al, 1986).

The greater prevalence of atypical naevi and the AMS phenotype in Australia implies that UVR can induce this phenotype. The odds ratios for melanoma in relation to common naevi were similar between the two countries, whereas for atypical naevi and the AMS phenotype there was evidence of larger odds ratios in England compared with Australia. This pattern would be expected if high-risk genetically determined AMS have been diluted in Australia by the presence of many individuals with 'sun-induced AMS', which may confer a lower risk of melanoma.

Twenty five per cent of all melanomas below the age of 50 were statistically attributable to the AMS phenotype in Australia compared with 16% in England, and the predictive value of the AMS phenotype decreased with age in both countries. The mean age of melanoma was in the early fifties in both countries and the AMS phenotype would be a poor predictor of melanoma in that older age group. For younger age groups, however, the presence of two or more atypical naevi or the presence of the AMS phenotype may be more powerful predictors of risk. Individuals with large numbers of atypical or common naevi should be especially targeted for self-examination and reduction of sun exposure in Australia. In the UK, the incidence of melanoma is much lower and the presence of the AMS phenotype accounts for a lower proportion of melanoma, so the public health gain from measures targeted at this group would be less. However, this phenotype is more predictive of an increased risk of melanoma in the UK than in Australia. Screening the UK population for the AMS phenotype is unlikely to significantly reduce melanoma mortality, but there may be a need for more public education emphasising the importance of the naevus phenotype as a risk factor for melanoma in the UK with a view to encouraging reduction of sun exposure and self-examination (with self-selected screening) in the high-risk groups.

© Cancer Research Campaign 1998

This study supports the importance of UVR exposure in the expression of the naevus phenotype. As for many other cancers, gene environment interaction plays an important role in melanoma and the relative contribution of genetic factors and sun exposure in the causation of melanoma and the expression of the naevus phenotype needs to be further elucidated. Further advances in AMS family studies may lead to the discovery of one, or more likely several, genes responsible for naevus expression. The high penetrance of the AMS phenotype in melanoma families suggests that genetic factors are important (Greene et al, 1985; Newton et al, 1994). Furthermore, a UK study reported high concordance in naevi number in monozygotic twins compared with dizygotic twins, but the numbers were small (Easton et al, 1991). Despite different levels of sun exposure in Australia and the UK, the histological subtypes and sites of melanomas were very similar between countries, implying that sun exposure does not greatly influence part of the biological behaviour of the disease. The presence of atypical naevi or the AMS phenotype may be a useful screening tool for melanoma in younger age groups, especially in Australia. However, follow-up and intervention studies are needed to determine whether screening for young AMS individuals will be useful and, furthermore, whether reducing sun exposure in these high-risk groups will reduce melanoma incidence.

### ACKNOWLEDGEMENTS

We thank Jane Hickman for data entry and Elizabeth Pinney for helping to collect some of the data. We are also grateful to the dermatologists, surgeons, pathologists and general practitioners in Australia and England for allowing us to approach their patients for the study. This work was funded by the Imperial Cancer Research Fund, UK.

### REFERENCES

Armstrong BK, de Klerk NH and Holman CDJ (1986) Etiology of common acquired melanocytic naevi: Constitutional variables, sun exposure and diet. *J Natl Cancer Inst* **77**: 329–335

Armstrong BK and English DR (1988) The epidemiology of acquired melanocytic naevi and their relationship to malignant melanoma. *Pigment Cell* **9**: 27–47

Augustsson A, Stierner U, Rosdahl I and Suurkula M (1991) Common and dysplastic naevi as risk factors for cutaneous malignant melanoma in a Swedish population. *Acta Derm Venereol* **71**: 518–524

Bataille V, Newton Bishop JA, Sasieni P, Swerdlow AJ, Pinney E, Griffiths K and Cuzick J (1996) Risk of cutaneous melanoma in relation to the numbers, types and sites of naevi. A case-control study. *Br J Cancer* **73**: 1605–1611

Breslow NE and Day NE (1980) *Statistical Methods in Cancer Research* Vol 1. IARC Scientific Publication No 32: Lyon

Cannon-Albright LA, Goldgar DE, Meyer LJ Lewis C, Anderson DE, Fountain JW, Hegi ME, Wiseman RW, Petty EM and Bale AE, Olopade OI, Diaz MI, Kwiatkowski DJ, Piepkorn MW, Zone JJ and Skolnick MH (1992) Assignment of a locus for familial melanoma, MLM, to chromosome 9 p13–p22. *Science* **258**: 1148–1152

Cooke KR, Spears GFS and Skegg DCG (1985) Frequency of moles in a defined population. *J Epidemiol Community Health* **39**: 48–52

Easton DF, Cox GM, MacDonald AM and Ponder BAJ (1991) Genetic susceptibility to naevi. A twin study. *Br J Cancer* **64**: 1164–1167

Fritschi L, McHenry P, Green A, MacKie R, Green L and Siskind V (1994) Naevi in schoolchildren in Scotland and Australia. *Br J Dermatol* **130**: 599–603

Gallagher RP, McLean DI, Yang CP, Coldman AJ, Silver HK, Spinelli JJ and Beagrie M (1990) Suntan, sunburn and pigmentation factors and the frequency of acquired melanocytic nevi in children; Similarities to melanoma. The Vancouver Mole Study. *Arch Dermatol* **126**: 770–776

Green A, Bain C, MacLennan R and Siskind V (1986) Risk factors for cutaneous melanoma in Queensland. *Rec Res Cancer Res* **102**: 76–97

Greene MH, Clark WH Jr, Tucker MA, Kraemer KH, Elder DE and Fraser MC (1985) High risk of malignant melanoma in melanoma-prone families with dysplastic naevi. *Ann Int Med* **102**: 458–465

Greenland S (1987) Variance estimators for attributable fraction estimates, consistent in both large strata and spare data. *Stat Med* **6**: 701–708

Grob JJ, Gouvernet J, Aymar D, Mostaque A, Romano MH, Collet AM, Noe MC, Discontanzo MP and Bonerandi JJ (1990) Count of benign melanocytic nevi as a major indicator of risk for non-familial nodular and superficial spreading melanoma. *Cancer* **66**: 387–395

Grulich A, Bataille V, Swerdlow A, Newton-Bishop JA, Cuzick J, Hersey P and McCarthy WH (1996) A case-control study of melanoma in New South Wales, Australia. *Int J Cancer* **67**: 485–491

Harrison SL, MacLennan R, Speare R and Wronski I (1994) Sun exposure and melanocytic naevi in young Australian children. *Lancet* **344**: 1529–1532

Holly EA, Kelly JW, Shpall SN and Chiu SH (1987) Number of melanocytic nevi as a major risk factor for malignant melanoma. *J Am Acad Dermatol* **17**: 459–468

Holman CDJ and Armstrong BK (1984*a*) Pigmentary traits, ethnic origin, benign naevi and family history as risk factors for cutaneous malignant melanoma. *J Natl Cancer Inst* **72**: 257–266

Holman CDJ and Armstrong BK (1984*b*) Cutaneous malignant melanoma and indicators of total accumulated exposure to the sun. An analysis separating histogenic types. *J Natl Cancer Inst* **73**: 75–82

Kelly JW, Rivers JK, MacLennan R, Harrison S, Lewis AE and Tate BJ (1994) Sunlight: a major factor associated with the development of melanocytic nevi in Australian schoolchildren. *J Am Acad Dermatol* **30**: 40–48

Kopf AW, Lazar M, Bart RS, Dubin N and Bromberg J (1978) Prevalence of nevocytic nevi on lateral and medial aspects of arms. *J Dermatol Surg Oncol* **4**: 153–158

MacGeogh C, Newton Bishop JA, Bataille V, Bishop DT, Frischauf AM, Meloni R, Cuzick J, Pinney E and Spurr NK (1994) Genetic heterogeneity in familial malignant melanoma. *Human Mol Genet* **3**: 2195–2200

Mackie RM, English J, Aitchinson TC, Fitzsimons CP and Wilson P (1985) The number and distribution of benign pigmented moles (melanocytic naevi) in a healthy British population. *Br J Dermatol* **113**: 167–174

MacKie RM, McHenry P and Hole D (1993) Accelerated detection with prospective surveillance for cutaneous malignant melanoma in high risk groups. *Lancet* **341**: 1618–1620

MacLennan R, Green AC, McLeod GR and Martin NG (1992) Increasing incidence of cutaneous melanoma in Queensland, Australia. *J Natl Cancer Inst* **84**: 1427–1432

Newton Bishop JA, Bataille V, Pinney E and Bishop DT (1994) Family studies in melanoma: identification of the Atypical Mole Syndrome (AMS) phenotype. *Melanoma Research* **4**: 199–206

Nicholls EM (1973) Development and elimination of pigmented moles and the anatomical distribution of primary malignant melanoma. *Cancer* **32**: 191–195

Osterlind A, Tucker MA, Hou-Jensen K, Stone BJ, Engholm G and Jensen OM (1988) The Danish case control study of cutaneous malignant melanoma. Importance of host factors. *Int J Cancer* **42**: 200–206

Sigg C and Peloni F (1989) Frequency of acquired melanocytic nevi and their relationship to skin complexion in 939 schoolchildren. *Dermatologica* **179**: 123–128

Swerdlow AJ, English J, MacKie RM, O'Doherty CJ, Hunter JA, Clark J and Hole DJ (1986) Benign melanocytic naevi as risk factors for malignant melanoma. *Br Med J* **292**: 1555–1559

© Cancer Research Campaign 1998